# Coupled Dynamics of Vaccination Behavior and Epidemic Spreading on Multilayer Higher-Order Networks

**DOI:** 10.3390/e28020243

**Published:** 2026-02-20

**Authors:** Zhishuang Wang, Guoqiang Zeng, Qian Yin, Linyuan Guo, Zhiyong Hong

**Affiliations:** School of Electronics and Information Engineering, Wuyi University, Jiangmen 529020, China; zswang@wyu.edu.cn (Z.W.); zgqcay0214@163.com (G.Z.); gly_wyu@126.com (L.G.); hongmr@163.com (Z.H.)

**Keywords:** higher-order networks, vaccination behavior, epidemic spreading, coevolutionary dynamics

## Abstract

Vaccination behavior and epidemic spreading are strongly intertwined processes, and their coevolution is often shaped by both individual decision-making and social interactions. However, most existing studies model such interactions at the pairwise level, overlooking the potential impact of higher-order social influence arising from group interactions. In this work, we develop a coupled vaccination–epidemic spreading model on multilayer higher-order networks, where vaccination behavior evolves on a simplicial complex and epidemic propagation occurs on a physical contact network. The model incorporates imperfect vaccine efficacy, allowing vaccinated individuals to become infected, and introduces a hybrid vaccination strategy that combines rational cost–benefit evaluation with social influence from both pairwise and higher-order interactions, as well as negative effects induced by vaccine failure. By constructing the coupled dynamical equations, we analytically derive the epidemic outbreak threshold and elucidate how higher-order interactions, behavioral responses, and vaccine-related parameters jointly affect epidemic dynamics. Numerical simulations on networks with different structural properties validate the theoretical results and reveal pronounced structure-dependent effects. The results show that higher-order social interactions can significantly reshape vaccination behavior and epidemic prevalence, while network heterogeneity and vaccine imperfection play crucial roles in determining the outbreak threshold and steady-state infection level. These results emphasize the necessity of incorporating higher-order interactions together with realistic vaccination behavior into epidemic modeling and offer new insights for the design of effective vaccination strategies.

## 1. Introduction

The emergence and recurrence of epidemics have long posed severe risks to public health and socioeconomic stability [1]. Understanding how epidemics spread within complex populations has therefore become a central topic in statistical physics and network science [2,3]. Classical epidemic models, such as the susceptible–infected–susceptible (SIS) and susceptible–infected–recovered (SIR) frameworks, have provided fundamental insights into the macroscopic laws governing disease transmission. However, real-world disease dynamics are not solely driven by biological contagion; they are also profoundly shaped by human behavioral responses [4,5]. Among these, vaccination remains the most effective and sustainable measure for epidemic control, yet individuals’ decisions to vaccinate are rarely uniform or purely rational. Instead, they emerge from dynamic interactions between personal risk perception, social influence, and collective behaviors within a population [6,7]. Consequently, vaccination decisions and disease transmission form a feedback loop, in which epidemic prevalence affects vaccination willingness while vaccination coverage reshapes epidemic evolution, highlighting the need for coevolutionary epidemic modeling.

A substantial body of prior work examining the coupled dynamics of vaccination behavior and disease propagation is built upon traditional complex networks, where contagion and behavioral influence are both modeled as pairwise interactions between individuals [8,9,10,11,12]. Although these frameworks help explain the interplay between decision-making and epidemic outcomes, they treat social processes as binary contacts, neglecting the collective and context-dependent nature of human behavior. In reality, vaccination decisions are rarely made in isolation; they are shaped within social groups—families, workplaces, or online communities—where discussions, consensus formation, and peer pressure play decisive roles [13]. Consequently, pairwise-based models fail to capture nonlinear reinforcement and cooperative effects that emerge when multiple peers simultaneously influence an individual’s perception and choices, leading to an incomplete representation of behavioral diffusion and its feedback on epidemic dynamics [14]. To address these conceptual limitations, recent research has begun to incorporate higher-order network frameworks, such as hypergraphs and simplicial complexes, which extend traditional pairwise representations by allowing interactions among multiple nodes within a single event [15,16,17]. Such higher-order structures provide a more faithful characterization of social contagion processes characterized by group reinforcement, threshold effects, and cooperative activation. In such systems, the collective behavior of small groups can substantially alter macroscopic epidemic outcomes, reshaping transmission thresholds, steady states, and critical phenomena [18,19]. Incorporating higher-order interactions into coevolutionary models helps reveal how group mechanisms shape individual decisions and disease propagation.

Within the framework of traditional multiplex networks, extensive efforts have been devoted to modeling the coupled dynamics between vaccination behavior and epidemic spreading by explicitly incorporating information diffusion, behavioral responses, and policy interventions [20,21,22,23,24,25,26,27]. For instance, Kabir et al. proposed a two-layer SIR/V-UA framework on heterogeneous networks that couples awareness diffusion with voluntary vaccination games, demonstrating that information spreading under different risk-assessment strategies can enhance vaccination coverage, raise epidemic thresholds, and effectively suppress outbreaks, especially in scale-free networks [28]. Li et al. further investigated an evolutionary vaccination game formulated within multiplex network structures by integrating information dissemination with epidemic spreading, revealing that the effect of information diffusion on vaccination uptake and epidemic control depends jointly on vaccination costs, network structural features, and different phases of system evolution, and that increased information transmission does not necessarily promote vaccination [29]. Motivated by the COVID-19 Omicron variant, Luo et al. constructed a multilayer heterogeneous network framework to capture the coupled dynamics of information dissemination, behavioral adaptation, and epidemic propagation, and systematically analyzed how intra-layer parameters and inter-layer coupling strengths affect epidemic size and healthcare burden [30]. From a policy-oriented perspective, Wu et al. constructed a multilayer coupled network model incorporating government publicity, vaccination incentives, and disease intervention measures, and, within the microscopic Markov chain (MMC) approach, showed that coordinated multi-level policies can significantly increase epidemic thresholds and reduce infection prevalence [31]. In addition, Zheng et al. extended the multilayer modeling paradigm by considering negative information diffusion and immunization behavior in a dynamic multilayer framework, demonstrating that official clarification, individual discernment ability, and physical fitness play crucial roles in suppressing misinformation spread and mitigating epidemic risk [32].

Beyond traditional multilayer network frameworks based on pairwise interactions, a growing body of research has highlighted the essential contribution of higher-order interactions to the formation of the coupled dynamics of information–disease spreading [33,34,35]. Fan et al. developed an information–disease coevolutionary spreading framework on multilayer simplicial complexes and demonstrated that higher-order interactions represented by 2-simplexes in virtual social networks can significantly enhance information diffusion, raise epidemic outbreak thresholds, and effectively suppress disease spreading under certain conditions [36]. Wang et al. investigated the interplay between simplicial awareness contagion and epidemic spreading on time-varying multiplex networks, demonstrating that group-level reinforcement significantly influences epidemic thresholds and spreading dynamics [37]. Similarly, Zhu et al. investigated a coupled information–disease model in which the information layer exhibits higher-order clique structures, showing that increased higher-order clustering leads to higher epidemic thresholds and lower steady-state infection levels [38]. From a different perspective, Song et al. studied asymmetric coupled dynamics on higher-order multilayer networks by introducing non-pairwise infection mechanisms and multiple information-spreading strategies, revealing that higher-order infection processes generally promote disease transmission, whereas appropriate information-spreading strategies, particularly degree-unbiased diffusion, can counteract this effect and most effectively suppress epidemic outbreaks [39]. Furthermore, Liu et al. introduced a mechanism for cross-validating information from multiple sources driven by group-based interactions in a multilayer higher-order network framework and showed that non-pairwise interactions spanning both the information and physical layers jointly regulate propagation dynamics, with information verification from the physical layer playing a more prominent role in reducing steady-state infection levels [40]. Collectively, these studies indicate that higher-order network structures fundamentally reshape the coevolutionary relationship between information spreading and disease dynamics, giving rise to richer nonlinear effects and offering new mechanisms for epidemic suppression that cannot be captured by classical network models restricted to pairwise interactions.

Despite the growing interest in higher-order interactions in coupled information–disease spreading, most existing studies focus primarily on how higher-order structures reshape information diffusion and epidemic transmission, while how non-pairwise interactions influence the coevolution of vaccination decisions and disease transmission remains largely unexplored. In real social systems, individuals’ vaccination intentions are not only influenced by each neighbor independently, but are often shaped by collective interactions involving multiple neighbors simultaneously. For example, group discussions among family members, colleagues, or online communities can generate social reinforcement effects that fundamentally differ from the superposition of pairwise influences. Traditional network models based solely on dyadic interactions are therefore insufficient to capture such group-induced behavioral responses. Higher-order complex networks based on simplicial complexes provide a natural framework to represent both individual-level influences and multi-individual interactions within a unified structure, enabling a more accurate description of vaccination decision-making processes. Motivated by this observation, it is necessary to investigate the coevolutionary dynamics of vaccination behavior and disease transmission within a two-layer network with non-pairwise interactions, so as to elucidate how non-pairwise interactions in the behavioral layer reshape epidemic thresholds, steady states, and coevolutionary dynamics, and to gain deeper theoretical insights into collective vaccination-driven epidemic control.

The primary contributions of this work are outlined below. First, we establish a vaccination–epidemic coevolutionary framework within a two-layer network characterized by higher-order interactions based on simplicial complexes, where vaccination is assumed to be imperfect, allowing vaccinated individuals to remain susceptible to infection with a reduced probability. Second, we introduce a mixed vaccination strategy that simultaneously accounts for vaccination cost and neighbor influence, where the latter incorporates both pairwise interactions and higher-order social interactions, while explicitly considering the negative impact of ineffective neighbors. Third, we formulate the coupled dynamical equations governing vaccination behavior and epidemic spreading, perform theoretical analysis, and derive the epidemic outbreak threshold associated with the proposed framework. Finally, extensive numerical experiments are conducted to validate the theoretical predictions and to further investigate the coupled dynamical behavior of vaccination adoption and disease transmission.

The rest of the paper is structured as follows. Section 2 introduces the coupled vaccination–epidemic model on a two-layer higher-order network. Section 3 outlines the vaccination decision strategy. Section 4 provides the theoretical analysis and derives the epidemic outbreak threshold. Section 5 reports numerical simulations and analyzes the principal findings. Finally, Section 6 provides concluding remarks.

## 2. Two-Layer Coupled Spreading Model

**Figure 1:**
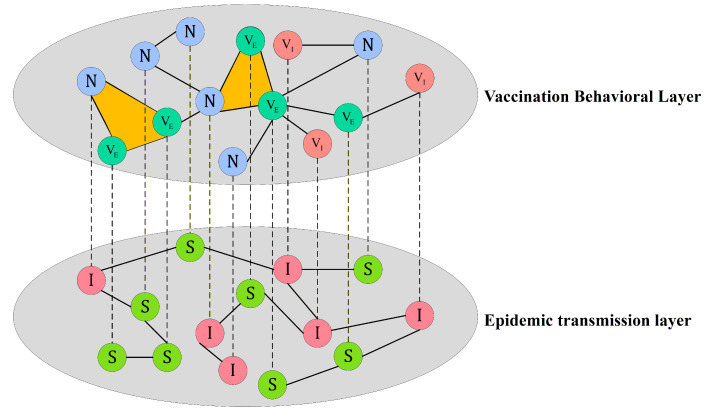
Schematic of the two-layer coupled model. The upper layer is a higher-order vaccination behavioral network with states *N*, $V_{E}$, and $V_{I}$, incorporating both pairwise links and 2-simplex interactions. The lower layer is a pairwise epidemic transmission network with states *S* and *I*. Interlayer links connect the same individual across layers, capturing the coevolution of vaccination behavior and disease spreading.

To elucidate the coupled dynamics between vaccination behavior and epidemic spreading in social systems with higher-order interactions, we construct a dual-layer network model as illustrated in Figure 1. These two layers represent interaction processes of different nature while sharing the same set of nodes, such that each node corresponds to the same individual in both layers. The upper layer corresponds to the vaccination behavioral diffusion layer, which characterizes the evolution of individuals’ vaccination intentions. This layer is modeled as a higher-order network based on simplicial complexes, incorporating both traditional pairwise links and group-level interactions formed by three-node 2-simplices. Individuals in this layer can be in one of three states: non-vaccinated (*N*), vaccinated with effective immunity ($V_{E}$), and vaccinated with ineffective immunity ($V_{I}$). The lower layer corresponds to the epidemic transmission layer, which characterizes the disease propagation dynamics on a conventional contact network with only pairwise interactions. Individuals in this layer may exist as either susceptible (*S*) or infected (*I*). Nodes in the two layers are connected through interlayer coupling links, indicating that vaccination decisions and disease states coexist and coevolve within the same individual. This structure allows us to investigate the interplay between vaccination behavior and disease transmission.

### 2.1. Vaccination Behavioral State Transitions

**Figure 2:**
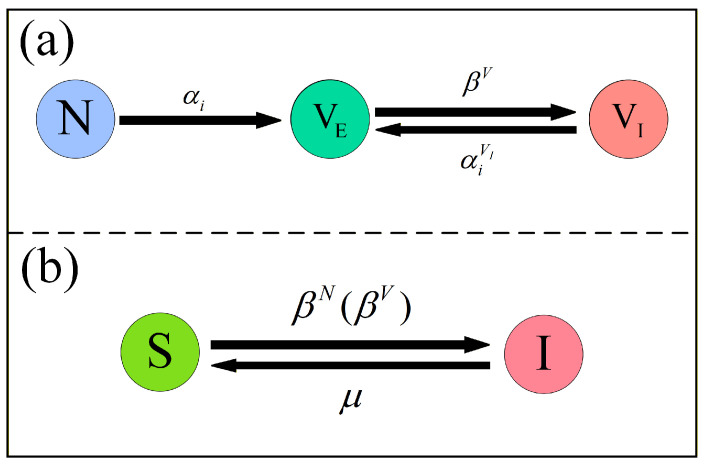
State transition diagrams of the coupled model. (**a**) Vaccination behavioral dynamics, where individuals move from *N* to $V_{E}$ with probability $\alpha_{i}$ and cycle between $V_{E}$ and $V_{I}$ with probabilities $\beta^{V}$ and $\alpha_{i}^{V_{I}}$. (**b**) SIS disease dynamics in the epidemic layer with infection probabilities $\beta^{N}$ and $\beta^{V}$ and recovery probability μ.

In the vaccination behavioral layer, unvaccinated individuals are initially in state *N*, and once they complete the first vaccination, their state changes to vaccinated with effective immunity $V_{E}$. Subsequently, the vaccination state evolves cyclically only between $V_{E}$ and $V_{I}$, while the unvaccinated state *N* does not participate in this cycle and serves solely as the entry state of the vaccination process, as illustrated in Figure 2a. Specifically, an individual *i* in state *N* chooses to receive vaccination with probability $\alpha_{i}$, and upon successful vaccination, transitions to state $V_{E}$. Considering the imperfect efficacy of vaccines, individuals in state $V_{E}$, although partially protected, still face a nonzero risk of infection. If such an individual becomes infected in the epidemic transmission layer, this indicates vaccine failure, and the corresponding state in the vaccination behavioral layer changes from $V_{E}$ to vaccinated with ineffective immunity $V_{I}$, with transition probability denoted by $\beta^{V}$. Furthermore, individuals in state $V_{I}$ may choose to be vaccinated again after recovering in the epidemic layer. This assumption reflects realistic behavioral responses observed in recurrent epidemic settings. For example, in seasonal influenza, individuals who become infected despite prior vaccination often choose to be vaccinated again in subsequent seasons to enhance protection, especially when the perceived infection risk remains high. At the same time, experiencing vaccine failure may reduce confidence in vaccination. To capture this dual behavioral response, we introduce a vaccine distrust parameter δ∈[0, 1], such that the probability of revaccination is correspondingly reduced to

(1) $$\alpha_{i}^{V_{I}}=\left(1-\delta \right)\alpha_{i}.$$

Once an individual decides to receive revaccination, the vaccination state transitions from $V_{I}$ back to $V_{E}$. This mechanism characterizes the evolutionary process of “initial vaccination, vaccine failure, and revaccination” in the vaccination behavioral layer and provides the foundation for constructing the coupled dynamical equations of vaccination behavior and epidemic spreading. The main symbols and parameters used throughout the manuscript are summarized in Table 1 for ease of reference.

### 2.2. Epidemic State Transitions

Within the disease transmission layer, the disease dynamics follow the classical SIS model, in which individuals continuously switch between state *S* and state *I*, as illustrated in Figure 2b. Specifically, susceptible individuals can acquire infection through contacts with infected neighbors, while infected individuals undergo recovery and return to the susceptible class with probability μ. Importantly, the infection risk of an individual is regulated by its vaccination status, reflecting the regulatory effect of vaccination behavior on epidemic spreading. For individuals in the non-vaccinated state *N*, the probability of transitioning from *S* to *I* is set to $\beta^{N}=\beta$, where β denotes the baseline transmission probability on the contact network. For vaccinated individuals with effective immunity, the infection risk is reduced due to vaccine protection, and the corresponding infection probability is given by $\beta^{V}=\gamma \beta$, where γ∈[0, 1] is the reduction factor, and smaller values of γ indicate stronger vaccine efficacy. In contrast, vaccinated individuals with ineffective immunity experience the same infection probability as non-vaccinated individuals, namely β. This state transition mechanism captures the interdependence between vaccination status and disease transmission and provides the basis for constructing the coupled dynamical equations of the model.

## 3. Vaccination Decision-Making Strategy

In the vaccination behavioral layer, individuals make vaccination decisions based on their circumstances and surrounding environment. Specifically, individuals can obtain information about the infection status and vaccination status of their neighbors, thereby perceiving potential infection risks and protective benefits. On this basis, individuals determine their vaccination strategies by weighing the costs associated with vaccination against the expected benefits. Moreover, individuals’ vaccination decisions are influenced not only by vaccinated neighbors through pairwise interactions, but also by higher-order interaction effects arising from the simultaneous influence of multiple vaccinated neighbors. Within the simplicial-complex-based higher-order network structure, such group-level social influence is captured by 2-simplexes, enabling individuals to account for the synergistic effects of multiple neighbors when making vaccination decisions. These assumptions provide the foundation for constructing a mixed vaccination strategy that incorporates vaccination cost and neighbor influence in the subsequent analysis.

### 3.1. Cost-Based Vaccination Decision

To model individual vaccination decisions, we adopt a rational decision-making framework inspired by evolutionary game theory. The core idea is that individuals evaluate and compare the expected costs associated with vaccinating and not vaccinating, and tend to choose the option with the lower expected cost. Such a mechanism captures self-interested behavior under perceived infection risk while allowing for bounded rationality at the population level.

For simplicity, the cost induced by infection, including medical expenses and productivity loss, is normalized to unity. The direct cost of vaccination is denoted by *c*, where 0<c<1. Here, the infection cost is normalized to unity for modeling convenience, and vaccination is assumed to incur a lower but non-negligible cost. In practice, vaccination usually involves expenses, time, and perceived risks, whereas infection may lead to medical costs, productivity loss, and health damage; therefore, its overall cost is typically higher. This relative-cost setting is widely adopted in evolutionary vaccination and epidemic–behavior models to capture individuals’ cost–benefit decision-making under infection risk. When an individual *i* chooses vaccination at time step *t*, the expected cost consists of the vaccination cost and the residual infection risk due to imperfect vaccine efficacy. Accordingly, the expected cost of vaccination is defined as

(2) $$C_{i}^{V}\left(t\right)=-c-\left[1-q_{i}^{V}\left(t\right)\right],$$

where $q_{i}^{V}\left(t\right)$ represents the probability that a vaccinated individual *i* remains free from infection at time *t*. The term $1-q_{i}^{V}\left(t\right)$ thus quantifies the residual infection risk after vaccination.

If individual *i* remains unvaccinated at time *t*, the expected cost arises solely from the infection risk, which is given by

(3) $$C_{i}^{N}\left(t\right)=-\left[1-q_{i}^{N}\left(t\right)\right],$$

where $q_{i}^{N}\left(t\right)$ corresponds to the probability that an unvaccinated individual avoids infection at time *t*. Consequently, $1-q_{i}^{N}\left(t\right)$ measures the infection risk faced by an unvaccinated individual. These definitions imply that vaccination decisions are directly influenced by the perceived infection risks under different behavioral choices, in agreement with empirical and theoretical studies showing that risk perception plays a key role in vaccination uptake.

To translate cost comparison into a probabilistic vaccination decision, we employ the Fermi updating rule. Specifically, the likelihood that node *i* chooses vaccination at time *t* is defined as

(4) $$v_{i}^{c}\left(t\right)=\frac{1}{1+\exp\;\left[-\frac{C_{i}^{V}\left(t\right)-C_{i}^{N}\left(t\right)}{K}\right]},$$

where K>0 serves as a noise parameter characterizing how responsive individuals are to cost differences. Smaller values of *K* correspond to highly rational decision-making, in which individuals almost always select the lower-cost option, whereas larger values of *K* introduce stronger randomness, accounting for bounded rationality, imperfect information, or unmodeled psychological factors.

### 3.2. Neighbor-Induced Social Influence

**Figure 3:**
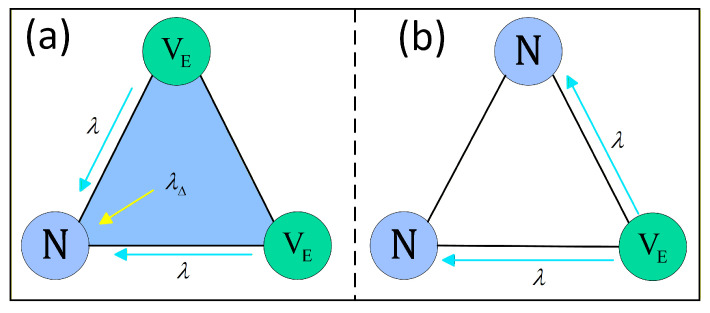
Schematic illustration of the activation condition for higher-order interactions based on a 2-simplex. (**a**) Higher-order influence is activated only when two neighbors within the simplex are in the effective vaccination state $V_{E}$. (**b**) If this condition is not satisfied, higher-order interaction does not occur and only pairwise influence is present.

Beyond cost-based rational decision-making, social influence at the group level constitutes a key driving force in the evolution of vaccination behavior. In social networks, individual decisions are shaped by neighbor influence, including peer pressure and group effects. To capture these complex mechanisms, the present model goes beyond traditional frameworks that consider only pairwise interactions and incorporates higher-order interaction structures in the vaccination behavioral layer. Specifically, 2-simplexes are introduced to describe group reinforcement effects arising from the simultaneous influence of multiple neighbors. The social influence mechanism thus operates at two complementary levels: pairwise influence and higher-order influence. On the one hand, unvaccinated individuals are directly affected by effectively vaccinated neighbors through network edges. On the other hand, as illustrated in Figure 3a, when two nodes within the same 2-simplex are in the effective vaccination state, the simplex itself generates a nonlinear group reinforcement effect, which promotes vaccination of the remaining unvaccinated node with an additional probability. Notably, this higher-order effect is subject to a strict activation condition: if only one vaccinated node is present within the simplex, as shown in Figure 3b, the higher-order interaction is not activated, and vaccination behavior spreads solely through conventional pairwise influence.

Based on the above description, we next quantify the probability that an individual is influenced by neighboring vaccinated individuals through pairwise and higher-order interactions. Specifically, we first define the probability that individual *i* is not affected by any neighboring individual through pairwise interactions at time *t*, denoted by $\phi_{i}^{\mathrm{edg}}\left(t\right)$, as well as the probability that individual *i* is not affected by any higher-order influence arising from 2-simplexes, denoted by $\phi_{i}^{\mathrm{tri}}\left(t\right)$. These probabilities are given by

(5) $$\left\{\begin{array}{cc} \phi_{i}^{\mathrm{edg}}\left(t\right) & =\prod_{j}\left[1-a_{ji}P_{j}^{V_{E}}\left(t\right)\lambda \right],\;\\ \phi_{i}^{\mathrm{tri}}\left(t\right) & =\prod_{l,j}\left[1-c_{lji}P_{j}^{V_{E}}\left(t\right)P_{l}^{V_{E}}\left(t\right)\lambda_{\Delta }\right], \end{array}\right.$$

where $P_{j}^{V_{E}}\left(t\right)$ denotes the probability that neighbor *j* is in the effective vaccination state $V_{E}$ at time *t*. The parameters λ and $\lambda_{\Delta }$ represent the basic influence strengths of pairwise and higher-order interactions, respectively. The term $a_{ji}$ corresponds to the element of the adjacency matrix in the vaccination behavioral layer, indicating whether a pairwise connection exists between individuals *j* and *i*. To characterize higher-order interactions, a simplicial adjacency matrix is further introduced, where $c_{lji}$ denotes the corresponding element associated with 2-simplex interactions involving individual *i*.

Accordingly, the probability that individual *i* is not exposed to any social influence promoting vaccination, including both pairwise and higher-order contributions, is given by $\phi_{i}\left(t\right)=\phi_{i}^{\mathrm{edg}}\left(t\right)\phi_{i}^{\mathrm{tri}}\left(t\right)$. Consequently, the probability that individual *i* experiences enhanced social influence toward vaccination adoption at time *t* is defined as $\omega_{1}\left(t\right)=1-\phi_{i}\left(t\right)$.

In addition to the positive reinforcement induced by effectively vaccinated neighbors, we further consider the negative influence exerted by neighbors in the ineffective vaccination state $V_{I}$. In realistic social settings, observing vaccination failure among surrounding individuals may undermine confidence in vaccine effectiveness and consequently reduce an individual’s willingness to vaccinate. To capture this weakening effect, we introduce a decay factor that characterizes the suppressive impact of $V_{I}$-state neighbors on vaccination intention. Specifically, the attenuation factor $\omega_{2}\left(t\right)$ is defined as

(6) $$\omega_{2}\left(t\right)=1-\frac{N_{i}^{V_{I}}\left(t\right)}{N_{i}^{V_{E}}\left(t\right)+N_{i}^{V_{I}}\left(t\right)},$$

where $N_{i}^{V_{E}}\left(t\right)$ and $N_{i}^{V_{I}}\left(t\right)$ denote the numbers of neighbors of individual *i* that are in the effective and ineffective vaccination states, respectively. Specifically, $N_{i}^{V_{E}}\left(t\right)$ and $N_{i}^{V_{I}}\left(t\right)$ are calculated within the vaccination behavioral layer based on the network neighborhood of node *i*. They represent the numbers of neighboring individuals directly connected to node *i* through pairwise links that are in states $V_{E}$ and $V_{I}$ at time *t*, respectively, excluding node *i* itself. These quantities are obtained according to the network structure of the behavioral layer and the state distribution of neighboring nodes. This formulation implies that the negative influence increases with the proportion of neighbors experiencing vaccine failure, thereby progressively weakening vaccination willingness.

By jointly considering the positive social reinforcement arising from group-level interactions and the negative feedback induced by ineffective vaccination experiences, the overall likelihood that individual *i* opts for vaccination driven by social influence at time *t* is expressed as

(7) $$v_{i}^{n}\left(t\right)=\omega_{1}\left(t\right)\;\omega_{2}\left(t\right),$$

where $\omega_{1}\left(t\right)$ captures the enhancement effect of pairwise and higher-order neighbor influence as defined previously. This multiplicative form reflects the combined and competing roles of reinforcing and suppressive social signals, enabling a balanced and realistic representation of neighbor-driven vaccination behavior.

### 3.3. Final Vaccination Decision Rule

Finally, we couple the two driving forces governing vaccination behavior, namely individual rational decision-making and neighbor-induced social reinforcement, to determine the final probability that an individual chooses vaccination. Specifically, the oaggregate vaccination likelihood for node *i* at time *t*, denoted by $\alpha_{i}\left(t\right)$, is formulated as a weighted linear mixture of the cost-based decision likelihood $v_{i}^{1}\left(t\right)$ and the neighbor-driven social influence probability $v_{i}^{2}\left(t\right)$,

(8) $$\alpha_{i}\left(t\right)=\left(1-\eta \right)\;v_{i}^{c}\left(t\right)+\eta \;v_{i}^{n}\left(t\right),$$

where the weighting parameter η(0≤η≤1) regulates the relative contribution of the two decision mechanisms to the vaccination decision-making process.

The parameter η provides a flexible way to interpolate between different behavioral regimes. When η=0, individuals behave as purely rational decision-makers whose vaccination choices are solely driven by cost–benefit evaluation. In contrast, when η=1, vaccination decisions are completely dominated by social influence from neighboring individuals, reflecting behavior primarily guided by collective reinforcement and social pressure. For intermediate values of η, individual decisions result from the combined effects of rational judgment and social influence. By systematically varying η, the proposed model allows us to explore how different decision-making modes shape the macroscopic dynamics of vaccination behavior and its interplay with epidemic spreading.

## 4. Theoretical Analysis

**Figure 4:**
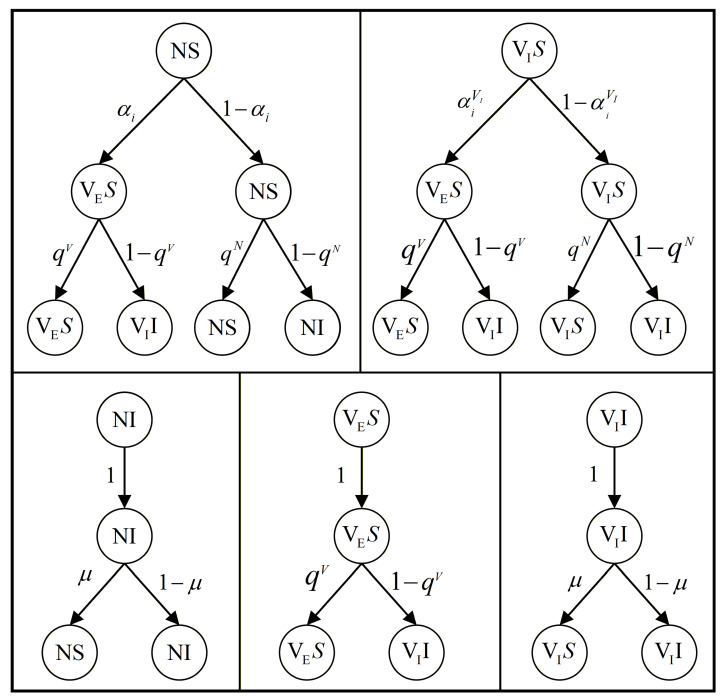
State-transition probability tree of the coupled vaccination–epidemic dynamics. The diagram illustrates the possible transitions among the five individual states within one time step, together with their corresponding transition probabilities, capturing the joint effects of vaccination decisions, vaccine efficacy, infection, and recovery processes.

Building upon the dynamical mechanisms presented in the preceding section, we first clarify the state space of individuals in order to perform a systematic theoretical analysis. In the present model, individuals can reside in five possible states under the coupled effects of vaccination behavior and epidemic spreading: unvaccinated and susceptible (NS), vaccinated with ineffective immunity and susceptible ($V_{I}S$), unvaccinated and infected (NI), vaccinated with effective immunity and susceptible ($V_{E}S$), and vaccinated with ineffective immunity and infected ($V_{I}I$). It should be noted that, due to the imperfect efficacy of vaccines, once a vaccinated individual in the effective immunity state becomes infected, the vaccine effectiveness is immediately lost. Consequently, the state $V_{E}I$ is not treated as an independent state in the model but is assumed to instantaneously transition to the vaccinated but ineffective and infected state $V_{I}I$. On this basis, we establish a state-transition probability tree to systematically characterize state changes across different compartments within one time step, as illustrated in Figure 4. This transition structure provides a clear theoretical framework for deriving the dynamical equations and epidemic thresholds using the MMC approach in the following analysis.

We further specify the probabilities that an node *i*, when vaccinated with effective protection or unvaccinated (or vaccinated but ineffective), avoids infection from all infected neighbors at time *t* as $q_{i}^{V}\left(t\right)$ and $q_{i}^{N}\left(t\right)$, respectively. These probabilities characterize the infection-avoidance process on the diseases spreading layer and are explicitly determined by the vaccination efficacy status of the individual. Under the independent transmission assumption, they are given by

(9) $$\left\{\begin{array}{c} q_{i}^{V}\left(t\right)=\prod_{j}\left[1-b_{ji}P_{j}^{I}\left(t\right)\beta^{V}\right],\;\\ q_{i}^{N}\left(t\right)=\prod_{j}\left[1-b_{ji}P_{j}^{I}\left(t\right)\beta^{N}\right], \end{array}\right.$$

where $b_{ji}$ denotes the element of the adjacency matrix of the epidemic transmission layer, indicating whether a contact exists between individuals *j* and *i*. The parameters $\beta^{V}$ and $\beta^{N}$ represent the infection probabilities associated with effectively vaccinated individuals and unvaccinated (or vaccine-ineffective) individuals, respectively. Here, the probability that neighbor *j* is infected is defined as $P_{j}^{I}\left(t\right)=P_{j}^{NI}\left(t\right)+P_{j}^{V_{I}I}\left(t\right)$, which includes all infected states regardless of vaccination history.

Based on Equation (9) and the state transition probability tree illustrated in Figure 4, we derive the coupled dynamical equations governing vaccination behavior and epidemic spreading within the framework of the MMC approach. In particular, for each discrete time step, the state transition of node *i* among the five possible states is jointly determined by its current state, vaccination decision, and the infection and recovery processes. By explicitly accounting for the distinct infection probabilities of unvaccinated individuals and vaccinated individuals with effective or ineffective protection in the disease spreading layer, together with the decision-making mechanism in the vaccination behavioral layer, the evolution equations of the state probabilities are formulated as

(10) $$\left\{\begin{array}{cc} P_{i}^{NS}\left(t+1\right) & =P_{i}^{NS}\left(t\right)\left(1-\alpha_{i}\left(t\right)\right)q_{i}^{N}\left(t\right)+P_{i}^{NI}\left(t\right)\mu ,\;\\ P_{i}^{NI}\left(t+1\right) & =P_{i}^{NS}\left(t\right)\left(1-\alpha_{i}\left(t\right)\right)\left(1-q_{i}^{N}\left(t\right)\right)+P_{i}^{NI}\left(t\right)\left(1-\mu \right),\;\\ P_{i}^{V_{E}S}\left(t+1\right) & =P_{i}^{NS}\left(t\right)\alpha_{i}\left(t\right)q_{i}^{V}\left(t\right)+P_{i}^{V_{E}S}\left(t\right)q_{i}^{V}\left(t\right)+P_{i}^{V_{I}S}\left(t\right)\alpha_{i}^{V_{I}}\left(t\right)q_{i}^{V}\left(t\right),\;\\ P_{i}^{V_{I}S}\left(t+1\right) & =P_{i}^{V_{I}S}\left(t\right)\left(1-\alpha_{i}^{V_{I}}\left(t\right)\right)q_{i}^{N}\left(t\right)+P_{i}^{V_{I}I}\left(t\right)\mu ,\;\\ P_{i}^{V_{I}I}\left(t+1\right) & =P_{i}^{NS}\left(t\right)\alpha_{i}\left(t\right)\left(1-q_{i}^{V}\left(t\right)\right)+P_{i}^{V_{E}S}\left(t\right)\left(1-q_{i}^{V}\left(t\right)\right)\;\\ \; & \;+P_{i}^{V_{I}S}\left(t\right)\alpha_{i}^{V_{I}}\left(t\right)\left(1-q_{i}^{V}\left(t\right)\right)+P_{i}^{V_{I}S}\left(t\right)\left(1-\alpha_{i}^{V_{I}}\left(t\right)\right)\left(1-q_{i}^{N}\left(t\right)\right)+P_{i}^{V_{I}I}\left(t\right)\left(1-\mu \right), \end{array}\right.$$

where each term represents the probability contribution from different initial states to the corresponding target state within one time step.

Accordingly, we proceed to examine the epidemic outbreak threshold associated with the proposed framework. When the system reaches a steady state, the proportions of individuals in the five possible states converge to constant values, and all terms in Equation (10) satisfy the steady-state condition

(11) $$P_{i}^{X}\left(t+1\right)=P_{i}^{X}\left(t\right)=P_{i}^{X},\;X\in \left\{NS,NI,V_{E}S,V_{I}S,V_{I}I\right\}.$$

Taking the limit t→∞, the epidemic threshold $\beta_{c}$ can be determined from the steady-state dynamical equations. As $\beta^{N}$ approaches the critical value $\beta_{c}$, the fraction of infected individuals in the system becomes vanishingly small. Under this assumption, the infection probability associated with node *i* can be approximated as $\varepsilon_{i}=P_{i}^{I}=P_{i}^{NI}+P_{i}^{V_{I}I}\ll 1$.

Under this near-threshold condition, higher-order terms of $P_{i}^{I}$ can be safely neglected. As a result, the infection-avoidance probabilities defined in Equation (9) can be linearized, yielding

(12) $$\left\{\begin{array}{c} q_{i}^{V}=\prod_{j}\left[1-b_{ji}P_{j}^{I}\left(t\right)\beta^{V}\right]\approx 1-\beta^{V}\sum_{j}b_{ji}\varepsilon_{j},\;\\ q_{i}^{N}=\prod_{j}\left[1-b_{ji}P_{j}^{I}\left(t\right)\beta^{N}\right]\approx 1-\beta^{N}\sum_{j}b_{ji}\varepsilon_{j}. \end{array}\right.$$

Substituting Equation (11) into Equation (12), the coupled system reduces to the following set of algebraic equations:

(13) $$\left\{\begin{array}{cc} P_{i}^{NS} & =P_{i}^{NS}\left(1-\alpha_{i}\right),\\ P_{i}^{V_{E}S} & =P_{i}^{NS}\alpha_{i}+P_{i}^{V_{E}S}+P_{i}^{V_{I}S}\alpha_{i}^{V_{I}},\\ P_{i}^{V_{I}S} & =P_{i}^{V_{I}S}\left(1-\alpha_{i}^{V_{I}}\right),\\ \mu \varepsilon_{i} & =P_{i}^{NS}\left(1-\alpha_{i}\right)\left(1-q_{i}^{N}\right)+P_{i}^{NS}\alpha_{i}\left(1-q_{i}^{V}\right)+P_{i}^{V_{E}S}\left(1-q_{i}^{V}\right)\\ \; & \;+P_{i}^{V_{I}S}\alpha_{i}^{V_{I}}\left(1-q_{i}^{V}\right)+P_{i}^{V_{I}S}\left(1-\alpha_{i}^{V_{I}}\right)\left(1-q_{i}^{N}\right), \end{array}\right.$$

where $\varepsilon_{i}=P_{i}^{NI}+P_{i}^{V_{I}I}$ represents the total probability that node *i* is infected.

Using the first three equations in Equation (13), together with the relations $\beta^{V}=\gamma \beta^{N}=\gamma \beta$, the last equation can be further simplified. Retaining only the linear terms of $\varepsilon_{i}$, we obtain

(14) $$\mu \varepsilon_{i}=P_{i}^{NS}\beta^{N}\sum_{j}b_{ji}\varepsilon_{j}+P_{i}^{V_{E}S}\beta^{V}\sum_{j}b_{ji}\varepsilon_{j}+P_{i}^{V_{I}S}\beta^{N}\sum_{j}b_{ji}\varepsilon_{j},$$

which admits a more concise representation given by

(15) $$\mu \varepsilon_{i}=\left(P_{i}^{NS}+\gamma P_{i}^{V_{E}S}+P_{i}^{V_{I}S}\right)\beta \sum_{j}b_{ji}\varepsilon_{j}.$$

Let the total fraction of individuals who have been vaccinated with effective protection be denoted by $P_{i}^{V_{E}}=P_{i}^{V_{E}S}$, and the total fraction of individuals whose vaccination has become ineffective be denoted by $P_{i}^{V_{I}}=P_{i}^{V_{I}S}$. Since the probabilities of all five states satisfy the normalization condition $P_{i}^{NS}+P_{i}^{NI}+P_{i}^{V_{E}S}+P_{i}^{V_{I}S}+P_{i}^{V_{I}I}=1$, the fraction of unvaccinated susceptible individuals can be approximated as

(16) $$P_{i}^{NS}\approx 1-P_{i}^{V_{E}}-P_{i}^{V_{I}}.$$

Substituting Equation (16) into Equation (15), the linearized infection dynamics can be further simplified as

(17) $$\mu \varepsilon_{i}=\left[1+\left(\gamma -1\right)P_{i}^{V_{E}}\right]\beta \sum_{j}b_{ji}\varepsilon_{j}.$$

Equation (17) admits the following equivalent representation:

(18) $$\sum_{j}\left\{\left[1+\left(\gamma -1\right)P_{i}^{V_{E}}\right]b_{ji}-\frac{\mu }{\beta }\delta_{ij}\right\}\varepsilon_{j}=0,$$

where $\delta_{ij}$ denotes the entry of the identity matrix. Introducing the matrix H with entries $h_{ij}=\left[1+\left(\gamma -1\right)P_{i}^{V_{E}}\right]b_{ji}$, the existence of nontrivial solutions to Equation (18) reduces to an eigenvalue problem of matrix H. Denoting by $\Delta_{\max}\left(H\right)$ the largest eigenvalue of H, the critical condition for epidemic emergence of the model is finally obtained as

(19) $$\beta_{c}=\frac{\mu }{\Delta_{\max}\left(H\right)}.$$

From Equation (19), it is clear that $\beta_{c}$ is depends collectively on the structural properties of the disease-spreading layer, the fractions of effectively vaccinated individuals $P_{i}^{V_{E}}$, the recovery rate μ, and the infection attenuation factor γ. Conceptually, $\beta_{c}$ represents the minimum transmission probability required for sustained epidemic spreading in the coupled system. A larger recovery rate or higher proportion of effectively vaccinated individuals reduces the effective transmission capability, thereby increasing the epidemic threshold and making outbreaks less likely. In contrast, weaker vaccine protection or stronger structural heterogeneity in the contact network enhances transmission potential and lowers the threshold. This result illustrates how vaccination behavior, vaccine efficacy, and network structure jointly regulate epidemic emergence through their influence on the effective spreading matrix.

## 5. Numerical Simulation Results

In this section, we conduct numerical simulations to systematically analyze and validate the proposed coupled framework describing vaccination behavior and epidemic transmission. Specifically, we combine MMC iterative equations with Monte Carlo (MC) simulations to generate quantitative results, thereby verifying the theoretical analysis and further exploring the dynamical characteristics of the coupled propagation process. Regarding the network configuration, the vaccination decision layer is constructed using a random simplicial complex structure [41], with an average degree of 10 and, unless otherwise specified, two 2-simplices participated in by each individual. The disease transmission layer is modeled using both Erdős–Rényi (ER) and Barabási–Albert (BA) networks, each with an average degree of 6. For all simulation experiments, the network size is fixed at 1000 nodes. In addition, we define $\rho^{X}$ ($X\in \{I,S,V_{E},V_{I}\}$) as the density of individuals in state *X*. For all experiments, the initial conditions are set as $\rho^{I}=\rho^{V_{E}}=0.01$ and $\rho^{V_{I}}=0$, ensuring the comparability of simulation results across different scenarios.

**Figure 5:**
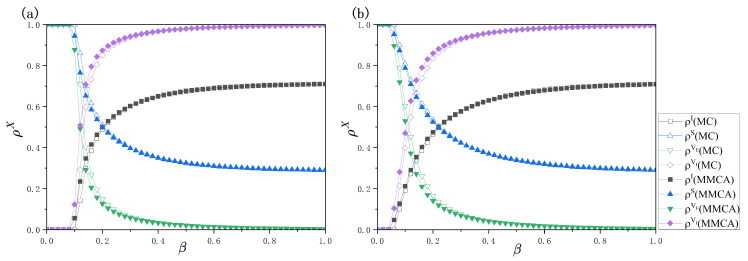
Steady-state densities of different states as functions of the transmission probability β. Panel (**a**) corresponds to the case where the epidemic transmission layer is an ER network, while panel (**b**) corresponds to a BA network. The model parameters are chosen as η=0.4, c=0.5, δ=0.4, γ=0.6, λ=0.4, $\lambda_{\Delta }=0.5$, μ=0.4, and K=0.5.

Based on the numerical simulation outcomes illustrated in Figure 5, the steady-state behavior of the system under different transmission probabilities β can be analyzed. Figure 5a,b depict the variations of the densities $\rho^{X}\;\left(X\in \left\{I,S,V_{E},V_{I}\right\}\right)$ as functions of β for different network structures. As β increases, the proportion of infected individuals $\rho^{I}$ grows monotonically, while the proportion of susceptible individuals $\rho^{S}$ decreases accordingly, indicating that a higher transmission probability significantly enhances disease spreading in the system. For all state densities, both the MMC approach and MC simulations are employed, and the comparison demonstrates a very good agreement between the two methods over the entire parameter range. In Figure 5a, the relative errors of $\rho^{I}$, $\rho^{S}$, $\rho^{V_{E}}$, and $\rho^{V_{I}}$ are 0.63%, 0.63%, 1.31%, and 1.31%, respectively, while in Figure 5b they are 0.45%, 0.45%, 1.19%, and 1.19%. These results confirm that the proposed MMC equations can accurately capture the dynamical characteristics of the coupled vaccination and epidemic spreading model.

Further inspection of Figure 5 shows that, regardless of whether an epidemic outbreak occurs, individuals eventually tend to choose vaccination. However, the dominant driving factors behind vaccination behavior differ markedly before and after the epidemic outbreak threshold. Below the threshold, vaccination decisions are mainly driven by social influence arising from the vaccination status of neighbors. In contrast, above the threshold, vaccination behavior is jointly driven by neighbor influence and the increasingly elevated infection risk, which together constitute the primary motivation for individuals to adopt vaccination. Moreover, when the transmission probability exceeds the outbreak threshold and continues to increase, the proportion of effectively vaccinated individuals $V_{E}$ decreases rapidly. This decline is primarily caused by the growing number of infected individuals, which leads to an increase in vaccine failure cases $V_{I}$ and thus exerts a stronger negative impact on vaccination willingness. In particular, under relatively high transmission probabilities, the fraction of effectively vaccinated individuals can become very small; as illustrated in Figure 5a, when β=0.6, $\rho^{V_{E}}$ is 0.0138, indicating that imperfect vaccination provides only limited protection in high-risk epidemic environments.

**Figure 6:**
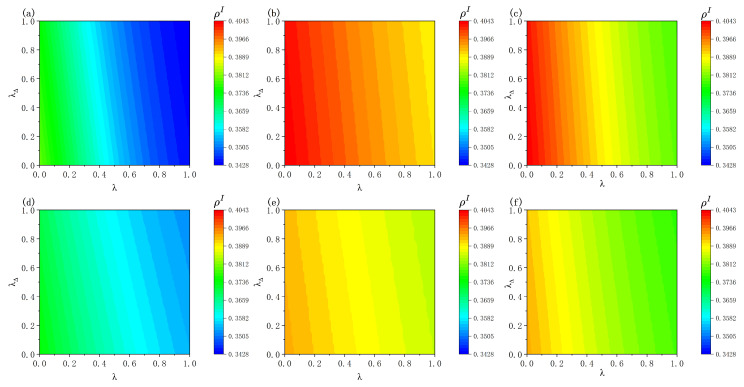
Influence of pairwise interaction strength λ, simplicial interaction strength $\lambda_{\Delta }$, and vaccine distrust level δ on the steady-state infected density $\rho^{I}$, with additional analysis of the effect of 2-simplex density. Panels (**a**–**c**) show results on ER networks and panels (**d**–**f**) on BA networks. Panels (**a**,**d**) correspond to δ=0.2, while panels (**b**,**c**,**e**,**f**) correspond to δ=0.5. Panels (**c**,**f**) consider a higher 2-simplex density, where each individual participates in four 2-simplices on average. Unless otherwise specified, the parameters are fixed as η=0.4, c=0.5, γ=0.6, μ=0.4, K=0.5, and $\beta^{N}=0.15$.

Figure 6 systematically illustrates the combined effects of the edge-based interaction strength λ and the higher-order simplicial interaction strength $\lambda_{\Delta }$ in the vaccination behavioral layer on individuals’ vaccination influence probabilities as well as the density $\rho^{I}$. Overall, for both ER and BA epidemic transmission networks, the steady-state infection density decreases to varying degrees as λ and $\lambda_{\Delta }$ increase. This indicates that both local social influence arising from pairwise neighbor interactions and collective reinforcement effects induced by higher-order group interactions can effectively enhance individuals’ vaccination willingness, thereby suppressing epidemic prevalence in the steady state. Further comparisons between Figure 6a,b, as well as between Figure 6c,e, reveal that under identical parameter settings, a larger vaccine distrust level δ leads to a higher infection density. This behavior is primarily driven by the underlying mechanism where increasing δ reduces individuals’ willingness to be revaccinated after experiencing vaccine failure, causing some individuals to remain in the vaccinated-but-ineffective state for longer periods. As a result, the suppressive influence of immunization actions upon the contagion process is weakened, yielding a higher steady-state infection level.

Further comparisons between Figure 6a,d, as well as between Figure 6b,e, reveal an interesting structure-dependent phenomenon. When δ=0.2, under otherwise identical conditions, the steady-state infection density in the BA epidemic transmission topology exhibits a marginally greater value compared to the ER structure. In contrast, when δ=0.5, the opposite pattern emerges, with the ER network exhibiting a higher infection density than the BA network. This reversal arises from the coupled effects of vaccine distrust and network topology. For smaller δ, the inhibitory effect of vaccine failure on subsequent vaccination willingness is limited, allowing the behavioral layer to maintain relatively high vaccination activity. Under these circumstances, the macroscopic infection level depends largely on the intrinsic spreading capability within the contagion layer, and the strong topological heterogeneity of the BA network leads to a higher infection density. However, for larger δ, vaccine failure significantly suppresses individuals’ revaccination willingness, causing some individuals to remain in the $V_{I}$ state for extended periods, and the role of network structure in modulating infection risk becomes more pronounced. Specifically, in BA networks, the higher infection risk associated with hub nodes encourages individuals to choose revaccination even after vaccine failure, partially mitigating the negative effects of vaccine distrust. In contrast, in ER networks, where infection risks are relatively homogeneous and weaker, they are insufficient to offset the deterrent effect of vaccine failure on vaccination decisions, resulting in a higher steady-state infection level.

Further comparison of Figure 6b,c, as well as Figure 6e,f, shows that as the average number of 2-simplices participated in by each individual increases, the steady-state infected density decreases overall, indicating that a higher density of higher-order interactions helps suppress epidemic spreading. This is because more 2-simplices imply stronger group-level behavioral reinforcement, which enhances individuals’ willingness to be vaccinated again and sustains immune behavior, thereby reducing the effective transmission probability and limiting the epidemic scale. A further comparison between Figure 6c,f reveals that when the pairwise interaction strength λ is large, increasing the average number of 2-simplices per individual leads to a more pronounced reduction in infection density when the lower layer is an ER network. In ER networks, where connections are relatively homogeneous, the reinforcement of vaccination behavior induced by higher-order group interactions can form a more consistent level of immunity at the individual level, thereby weakening the overall transmission process. In contrast, in BA networks with highly heterogeneous degree distributions, a few hub nodes dominate the spreading process; even though higher-order interactions enhance vaccination behavior, their suppressive effect on transmission is more easily offset by the strong spreading capability of highly connected nodes, resulting in a relatively smaller reduction in infection density.

**Figure 7:**
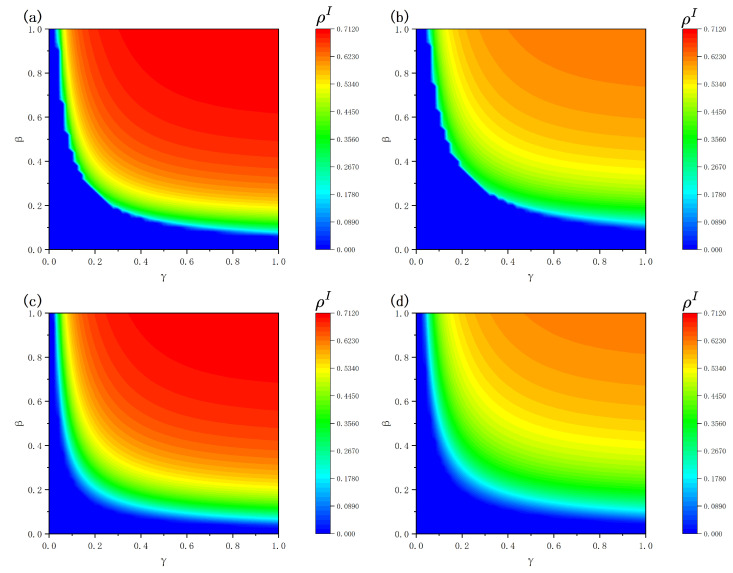
Steady -state infection density $\rho^{I}$ relative to changes in transmission probability β and the decay coefficient γ. Panels (**a**,**b**) correspond to ER networks in the disease-spreading layer, while panels (**c**,**d**) correspond to BA networks. Panels (**a**,**c**) are obtained with the recovery rate μ=0.4, whereas panels (**b**,**d**) correspond to μ=0.6. Other parameters are fixed as η=0.4, c=0.5, δ=0.4, λ=0.4, $\lambda_{\Delta }=0.5$, K=0.5.

The combined impact of infection likelihood β along with the attenuation factor γ on the steady-state infection density $\rho^{I}$ is demonstrated in Figure 7. Overall, across all panels, $\rho^{I}$ increases significantly with increasing β, indicating that the infection probability remains the dominant factor determining whether large-scale outbreaks occur and the resulting steady-state infection level. When β is small, the system remains in a low-infection or disease-free state; once β exceeds a critical region, the infection density rises rapidly and eventually converges to a relatively high steady level, exhibiting pronounced nonlinear transition behavior. Further examination of the role of γ reveals that, for a fixed β, the steady-state infection density increases monotonically with the attenuation factor. This implies that when the protective effect of vaccination against infection risk weakens (i.e., larger γ), infected individuals can more easily overcome protective barriers, thereby facilitating sustained disease transmission and leading to higher steady-state infection levels.

By comparing different recovery rates μ, it can be observed that increasing μ from 0.4 to 0.6 leads to an overall reduction in equilibrium contagion levels. Moreover, the low-infection region expands noticeably, and the critical transition boundary shifts toward larger values of β. This behavior reflects the effect of a higher recovery rate, which shortens the infectious period and suppresses transmission, requiring a larger infection probability β to sustain endemic states. Regarding the topological configuration of the disease-spreading layer, ER networks exhibit relatively homogeneous degree distributions, causing the transmission process to be primarily governed by the average connectivity. Consequently, the outbreak threshold is relatively concentrated, and once system parameters exceed the critical value, infections spread rapidly across the network, leading to a sharp increase in infection density near the threshold. In contrast, BA networks possess pronounced structural heterogeneity, where hub nodes can sustain localized transmission even under relatively low parameter values. This reduces the effective outbreak threshold and results in a smoother and more gradual increase in infection density as parameters vary.

**Figure 8:**
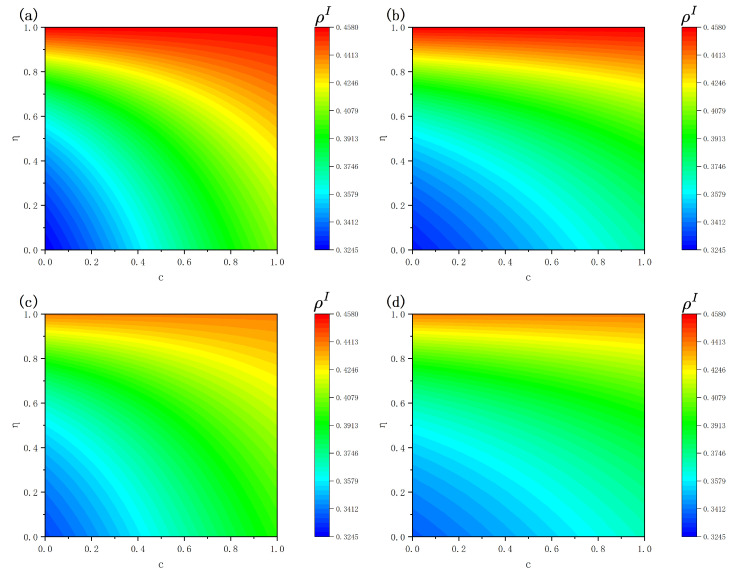
Effects of the weighting parameter η, vaccination cost *c*, and sensitivity factor *K* on the steady-state infection density $\rho^{I}$. Panels (**a**,**b**) correspond to ER networks in the disease transmission layer, while panels (**c**,**d**) correspond to BA networks. Panels (**a**,**c**) are obtained with K=0.5, whereas panels (**b**,**d**) are obtained with K=1. Values for other fixed parameters include δ=0.4, γ=0.6, λ=0.4, $\lambda_{\Delta }=0.5$, μ=0.4, and $\beta^{N}=0.15$.

From the overall trend observed in Figure 8, a positive correlation is observed between the equilibrium infection density $\rho^{I}$ and both the weighting parameter η and the expense of immunization *c*. Within the proposed model, η interpolates between rational decision-making and socially driven behavior: for small η, individuals tend to make vaccination decisions based primarily on cost–benefit evaluation, whereas increasing η shifts decision-making toward social influence from neighbors. When the vaccination cost *c* is low and rational evaluation dominates, individuals are more likely to vaccinate, thereby effectively suppressing disease transmission and maintaining a low steady-state infection level. As η increases, vaccination behavior becomes increasingly dependent on social feedback. In the presence of imperfect vaccine efficacy, infections within the population weaken positive social reinforcement for vaccination, reducing overall vaccination coverage and leading to higher steady-state infection densities. Meanwhile, a larger vaccination cost *c* further discourages vaccination under rational evaluation, and when combined with socially dominated decision-making, facilitates the persistence of higher infection levels.

A comparison between ER and BA networks further reveals a structure-dependent crossover behavior. When both η and *c* are small, the prevalence within ER topologies remains below the levels observed in BA networks; nevertheless, when η and *c* become large, the opposite trend emerges. For instance, when η=0.1 and c=0.1, the values of $\rho^{I}$ in Figure 8a and Figure 8c are 0.3352 and 0.3425, respectively, whereas when η=0.9 and c=0.6, the corresponding values are 0.4461 and 0.4296. This phenomenon indicates that under predominantly rational decision-making (small η), the structural heterogeneity of BA networks, especially the presence of hub nodes, facilitates disease persistence and results in higher infection levels. In contrast, when social influence dominates (large η) and vaccination costs are high, the amplification effect of social interactions around hub nodes in BA networks can promote more coordinated protective behavior, partially offsetting the negative effects of vaccine imperfection and high costs. ER networks, by contrast, lack such influential hubs, leading to more dispersed social influence and relatively higher infection levels in the high-η, high-*c* regime. Finally, by comparing Figure 8a,b as well as Figure 8c,d, it can be observed that a larger sensitivity factor *K* consistently results in reduced equilibrium infection levels under otherwise identical conditions, an impact that appears especially significant at lower η regimes. Such a finding suggests that when vaccination decisions are mainly driven by rational cost–benefit evaluation, a larger *K* amplifies individuals’ responsiveness to payoff differences, encouraging vaccination when costs are acceptable and thereby substantially suppressing disease transmission. As η increases and social influence becomes the dominant driver of behavior, the amplifying role of *K* on rational evaluation diminishes, resulting in a reduced difference in infection levels across different values of *K*.

**Figure 9:**
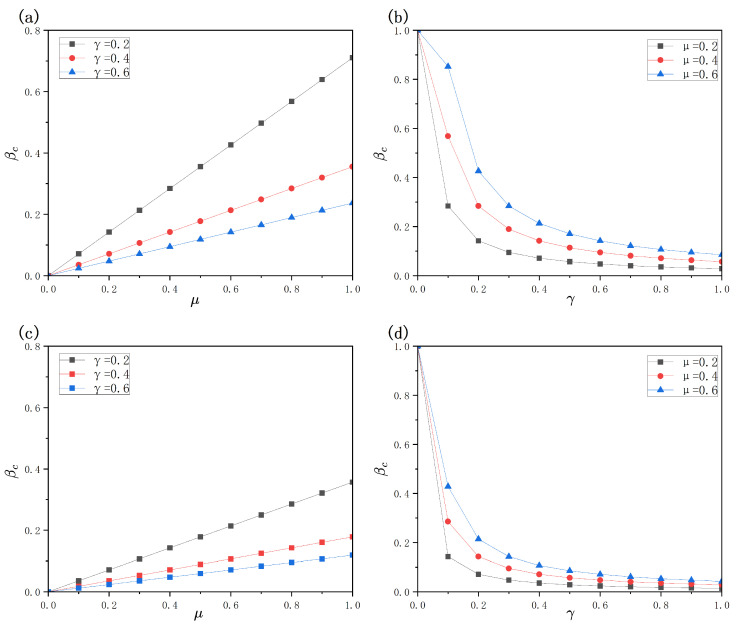
Effects of the contagion decay coefficient γ and the recovery rate μ on the threshold $\beta_{c}$. Panels (**a**,**b**) correspond to ER networks in the disease transmission layer, while panels (**c**,**d**) correspond to BA network structures. Specifically, panels (**a**,**c**) show the dependence of $\beta_{c}$ on μ across various γ levels, whereas panels (**b**,**d**) illustrate the dependence of $\beta_{c}$ on γ under several μ settings. The remaining parameters are fixed as η=0.4, c=0.5, δ=0.4, λ=0.4, $\lambda_{\Delta }=0.5$, and K=0.5.

From Figure 9a,c, it can plainly be seen that the critical spreading point $\beta_{c}$ increases monotonically alongside the healing rate μ. This phenomenon indicates that a higher recovery rate shortens the average infectious period of hosts, thus undermining the potent contagion potential of the infection. Consequently, a larger infection probability is required to sustain epidemic spreading, leading to an increased outbreak threshold. Further comparison between Figure 9a,c shows that, even under the influence of vaccination behavior, the outbreak threshold remains lower when the disease transmission layer is a BA network than when it is an ER network under the same recovery rate. This can be attributed to the pronounced structural heterogeneity of BA topologies, in which a few highly connected hub nodes exert a dominant role in the spreading process, enabling the disease to persist at lower infection probabilities and thus reducing the effective outbreak threshold.

From Figure 9b,d, it is observed that the critical spreading point $\beta_{c}$ undergoes a downward trend as the contagion attenuation factor γ increases. When γ=0, the vaccine remains fully effective and infection is strongly suppressed, so epidemic outbreaks can be completely prevented. As γ increases from small values, the vaccine failure effect becomes increasingly significant, allowing infections to break through vaccine protection more easily, which results in a rapid decrease of the outbreak threshold. However, when γ becomes sufficiently large, vaccine protection has already been substantially weakened, and further increases in γ have a diminishing marginal effect on disease transmission. Consequently, the corresponding decrease in the outbreak threshold becomes relatively moderate. These results indicate that the early-stage degradation of vaccine effectiveness plays a particularly pivotal part in governing the contagion spreading dynamics.

## 6. Conclusions

In this study, we explored the coevolution of vaccination behavior and disease spreading on two-layer higher-order networks by incorporating group-level social interactions into the behavioral layer. By modeling vaccination decisions as a combination of rational cost–benefit evaluation and neighbor-induced social influence on simplicial complexes, the proposed framework captures both individual decision-making and collective reinforcement effects that cannot be described by traditional pairwise network models. Imperfect vaccine efficacy and the negative impact of vaccine failure were further taken into account, allowing for a more realistic description of vaccination behavior evolution.

Based on the MMC approach, we derived the critical point for disease outbreaks and systematically analyzed how behavioral parameters, vaccine efficacy, and network topology jointly affect epidemic dynamics. Numerical simulations showed excellent agreement with the theoretical predictions and revealed several nontrivial phenomena. Higher-order interactions enhance vaccination willingness and suppress epidemic prevalence, whereas vaccine failure and distrust weaken this effect. Moreover, network structure plays a crucial role: networks with strong structural heterogeneity exhibit lower epidemic thresholds and smoother transition behaviors, while more homogeneous networks display sharper threshold effects and more abrupt changes in infection density.

Overall, our results highlight the importance of higher-order social structures in vaccination-driven epidemic dynamics and show that ignoring group-level interactions may limit the understanding of epidemic control. The proposed framework provides a flexible basis for exploring behavioral–epidemic coevolution in realistic social systems. Future work may extend this model to incorporate time-varying higher-order interactions, heterogeneous behavioral responses, or adaptive intervention strategies, thereby offering deeper insights into epidemic prevention and vaccination policy design.

## Figures and Tables

**Table 1:** Summary of main symbols.

Symbol	Meaning
λ	Pairwise interaction strength in the vaccination behavioral layer
$\lambda_{\Delta }$	Higher-order simplicial interaction strength
δ	Vaccine distrust level
η	Weight parameter in vaccination decision-making
*c*	Vaccination cost
γ	Infection attenuation factor due to vaccination
μ	Recovery rate in the epidemic layer
*K*	Noise parameter in the Fermi decision rule
β	Baseline infection probability
$\beta_{c}$	Critical infection probability
$\beta^{N}$	Infection probability for non-vaccinated individuals
$\beta^{V}$	Infection probability for vaccinated individuals
$\alpha_{i}$	Vaccination decision probability of individual *i*
$\alpha_{i}^{V_{I}}$	Revaccination probability of individual *i* in state $V_{I}$

## Data Availability

All data that support the findings of this study are included within the article.
